# Healthcare research and education: actively constructed research knowledge–a model for online systematic reviews and meta-analyses courses

**DOI:** 10.1186/s13104-025-07111-8

**Published:** 2025-03-10

**Authors:** Walid El Ansari, Kareem El-Ansari, Mohamed Hany

**Affiliations:** 1 College of Medicine, Ajman University, Ajman, United Arab Emirates; 2 Faculty of Medicine, St. George’s University, Saint George’s, Grenada; 3 Department of Surgery, Medical Research Institute, Alexandria University, Alexandria, Egypt; 4 Madina Women’s Hospital, Alexandria, Egypt

**Keywords:** Systematic review, Metabolic and Bariatric Surgery, Training, Online, Experiential

## Abstract

Employing a mixed-methods approach, this study: (a) scoped the literature for desirable characteristics of online learning environments; (b) analyzed and compared the available online systematic review and meta-analysis (SR/MA) courses; and (c) proposed an outline for a model online systematic review SR/MA course that is aligned with PRISMA principles, and (d) charted learning outcomes, module contents, interactive elements, feedback and module assessment, and course evaluation of the proposed course. The findings highlight the many distinctive aspects and design of the proposed model course, including its comprehensiveness, alignment with PRISMA principles, self-pace and self-direction, with high interactivity levels, augmented by the expert trainer guidance (‘human’ factor), and individualized feedback, and its conclusion with the incorporation of the learners in virtual research teams undertaking real SR/MA. The proposed model course integrates principles of problem-based learning, where in addition to the provided resources (interactive assignments and video tutorials), trainees will be actively conducting their own real SR/MA as part of the course, while receiving expert feedback to enhance learning outcomes. Assignments’ scores will be used to evaluate the learners’ proficiency levels, and to pinpoint strengths and areas requiring enhancement. Overall, the proposed model course stands out as an innovative initiative in online SR/MA training, offering a valuable and comprehensive online learner-centered, expert-guided, ‘hands-on’ educational approach to SR/MA training. This sets a benchmark for future online courses in research synthesis methodology within health, medical, surgical and related fields.

**Supplementary Information:**

The online version contains supplementary material available at 10.1186/s13104-025-07111-8.

## Background

Evidence-based practice is premised on the critical evaluations of literature comprising studies on a specific topic [1]. However, healthcare practitioners rarely have time to stay updated with the large, continuously developing evidence base [2], and the number of studies, sometimes with inconclusive, or inconsistent results, can confuse clinical decision-making [3, 4]. To provide patients with the best possible outcomes, healthcare decisions require updated, well-informed, high-quality research evidence [5, 6]. Therefore, an essential requirement of evidence-based care is the availability of systematic reviews and meta-analyses (SR/MA) that draw together, in an objective and systematic manner, all available evidence pertinent to a practice question [7]. SR are situated high on the evidence pyramid hierarchy [8]. Nonetheless, complexities are involved in searching, collecting, and analyzing research findings for robust scientific synthesis required for SR/MA.

First, the large numbers of scientific studies published [9] dictates that reviewers access the necessary resources [10, 11]. Material resources include costs of published articles, bibliometric software, and SR/MA management and data storage platforms [12]. Equally, the human resources required to process complex volumes of information need experts spanning several disciplines [13]. Hence, international multi-disciplinary research consortia [14] are now the norm [15, 16].

A challenge is that SR/MA necessitate specialized knowledge domains and experience to outline a protocol’s aims and methods, search and retrieve relevant studies, appraise quality, extract data, synthesize evidence, and interpret findings [17]. SR/MA are relatively scare because scholars concentrate on observational [18, 19] or experimental studies, due to their limited SR/MA experience [18]. The reduced interest in SR/MA is reflected in the training of future academics, focusing on techniques to collect and analyze new data rather than methodologies to synthesize evidence from published research [7].

Researchers lack the skills to conduct SR/MA [13]. Guidelines e.g., Preferred Reporting Items for Systematic Reviews and Meta-Analyses (PRISMA) [20] and instructions on how to conduct SR/MA were created to standardize the planning and execution of SR/MA [20]. However, these lack instructional and skills-focused content [21], and their application is not a substitute for structured training. Many SR/MA are “methodologically flawed, biased, redundant, or uninformative” [22], to the extent that journals require information about the PRISMA items when SR/MA are submitted to ensure rigor and quality. But despite mechanisms and regulators, authors display low adherence to checklists [23]. This suggests that researchers have a dearth of necessary SR/MA skills, knowledge, and methodological expertise. Such deficiencies, in turn, highlight the need for quality SR/MA training courses.

### Contemporary internet-based training: types, differences, and shortcomings

Online education can be ‘online’, ‘remote’, and ‘online distance’ learning [24]. While these might seem similar, differences exist in content, delivery methods, communication [24], and in relation to self-pace and self-direction, experiential interactivity, and human feedback system/s. For the first feature, most so called ‘online’ SR courses effectively lack the self-paced/directed aspects of online learning that provide learners autonomy to study at their own time/pace [25–27], rendering them effectively distance-learning courses transposed from traditional classroom format to online via videoconferencing. Some such courses might have even continued using their usual group-based teaching and exercise method/s, now transposed onto a meeting platform [24, 28].

As for the second aspect, many online SR/MA courses lack real interactivity [29], using the term ‘interactive’ to represent some online multiple-choice quizzes with answers presented through online resources e.g., live/video recorded lectures, reading materials, or tutorial sheets [30]. Although these are essential pedagogical backbones, alone they remain ineffective [31]. Interactive learning is when learners participate in activities and problems, reflect on the experience, identify acquired knowledge and skills, and apply them in the workplace [29]. This approach is also the cornerstone of problem-based learning, where learners actively participate in problem-driven scenarios [32] to develop knowledge, skills, teamwork, professionalism, critical thinking, and problem-solving abilities. Online learning incorporates interactive elements [33, 34], with practical assignments where learners apply their attained knowledge to real-life scenarios (i.e., experiential) [30]. However, very few online SR courses engage an interactive, experiential approach, confirming that available online SR/MA courses do not adhere to interactivity principles [35].

As for the third feature, suitable feedback system/s in online courses is critical [36, 37]. For instance, master's level learners valued individualized feedback, as it helped them better understand the topics and identify their strengths/weaknesses [38]. Constructive feedback positively enhances the efficacy of courses delivered via learning management systems (LMS, e.g., Moodle) [36]. However, providing individualized constructive feedback in a specialized SR/MA necessitates expert trainers with both discipline-specific knowledge and SR/MA skills. It remains unclear whether current online SR/MA courses provide expert-guided feedback on assignments. On the contrary, some courses design their assignments as multiple-choice quizzes/questions with short answers, essentially eliminating expert feedback which requires further resources (expert trainers, planning, time).

These above-mentioned knowledge gaps suggest a lack of self-paced, self-directed online SR/MA courses that offer learners experiential interactivity embedded in the training, from study design to data collection and synthesis of results, supplemented with individual feedback from expert trainer/s. Furthermore, to our knowledge, no SR/MA online courses seem available where trainers and learners possess expertise in the same subject, allowing the integration of exercises resembling real-life assignments grounded in the given field. These considerations acted as the driver for the development of a model SR/MA course described in this paper. Therefore, the specific aims of the present study were to:

1. Identify desirable features of online learning environments for SR/MA training.
2. Develop a contemporary, online, self-paced, self-directed, and interactive model for SR/MA training; identify and formulate its learning outcomes, module contents, interactive elements, assessment methods, and course outcome evaluation techniques.
3. Ensure alignment of the course modules and contents with PRISMA principles [20].
4. Appraise existing online interactive, self-paced, and self-directed SR/MA courses; extract their key features, and compare them to the proposed model course.
5. Propose course outcome evaluations.

The model in this paper would be important for educators and organizations embarking on creating online interactive, self-paced, and self-directed SR/MA courses that utilize hands-on exercises and one-on-one learner-expert trainer interactions.

## Materials and methods

**Fig. 1:**
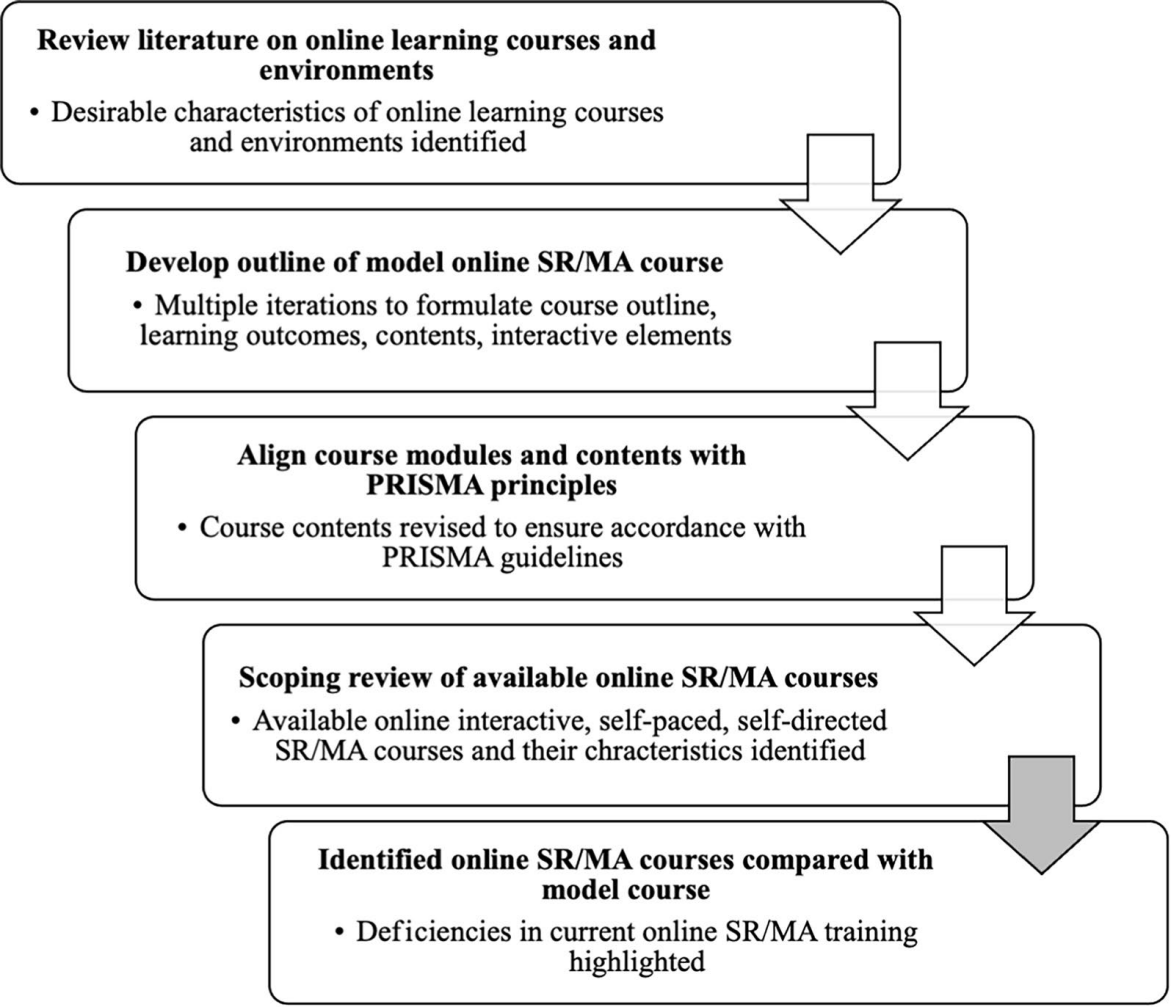
Steps to develop and refine a model online SR/MA course

This mixed-method study is a scoping review of the literature and educational websites, application of PRISMA guidelines, and formulation of a model course. Figure 1 depicts the steps undertaken to develop and refine a model online SR/MA course, Table 1 and Supplementary Table 1 illustrate the study objectives and their methods.

**Table 1:** Study objectives and corresponding methods employed

Objective	Method			
	Scoping		PRISMA Guidelines ^*b*^	Formulation/Brainstorming
	Literature review	Online courses		
1. Identify desirable features of online learning environments for SR/MA training^a^	✔			
2. Develop online, self-paced, self-directed, interactive, model for SR/MA training; design outline, learning outcomes, module contents, interactive elements, assessment			✔	✔
3. Align proposed course modules/contents with PRISMA principles			✔	
4. Identify and appraise current online SR/MA courses^a^, their strengths/limitations	✔	✔		
5. Formulate potential course outcome evaluation method/s	✔	✔		✔

^a^Included searching electronic databases, relevant documents, online resources, course outlines, reports, guidelines and other relevant online resources, Scoping review [39, 40]

^b^Guided by Page et al.[20]

## Results

### Desirable characteristics of online learning educational environments

Table 2 depicts selected advantageous characteristics of online courses. The many desirable features highlight the interacting nature that need to be incorporated for a robust online training model that includes self-paced and self-directed learning, interactive components, learning outcomes, module contents, assessment methods, and course evaluations. Collectively these perquisites suggest that a desirable environment contributes to focused learning experiences that cater for learning styles and schedules, where continuous evaluation contributes to ongoing improvement and quality.

**Table 2:** Desirable characteristics of online learning educational environments

Characteristic	Online learning
Description	Course content provided through digital platforms and channels, and utilizes interactive approach through embedded activities as fundamental part of the design and teaching strategy (66)
Location	Not location specific; Strictly online
Platform	Online learning environment or learning management system (67)
Learning material	Pre-recorded lectures/materials (some courses offer scheduled live sessions to achieve blended learning), interactive content (exercises, assignments, etc.) (68, 69)
Feedback	Active online feedback (70)
Learning time	Usually asynchronous; flexible (71)
Progression	Needs to have elements of self-pacing; self-direction is also advantageous
Interaction	Needs to engage learners by following interactive pattern and blended digital approach (72)
Communication	Interactions usually occur via online messaging platforms (allowing for immediate responses) or discussion boards and email (allowing for communication affected by geographical time differences) (73)
Requirements	Asynchronous nature can be more flexible; stable internet connection needed, but less stringent as learners must not be online at specific time, can download materials when they have access to online learning tools, and study offline if necessary
Intention	Learners receive study material and learn at their own pace, with interaction from peers and expert tutors
Assessment	Written assignment, online quizzes, online exams, fieldwork, digital projects (74)

### A model online SR/MA course: design, content, and interactive elements

The proposed course was inspired for busy medical/health students and clinicians worldwide interested in conducting SR/MA, who prefer to engage with the course on their own schedules (self-paced), and with content accessible on demand online (asynchronous delivery). To streamline the distribution of training materials and assignments, and seamless communication and feedback, the course utilizes Moodle^®^ LMS platform, recognized for its effectiveness in collaborative online learning and assessments [41], and enhanced teaching/learning experience [42]. It enables a clear overview of the course structure, supports synchronous and asynchronous one-on-one or group trainers-learners communication, providing a centralized hub for assignment submission and evaluation by expert trainers, with opportunity to resubmit assignments, if necessary, after receiving personalized feedback.

Table 3 shows the modular design of the SR/MA online course including module name, learning outcomes, and module characteristics. It comprises seven modules. The last module (Module 7) entails working within a team to prepare and write up a SR/MA manuscript. This represents a longer-term assignment, and initial preparation for this last module starts at beginning of the course and continues as an ongoing thread across all the course modules in a ‘building blocks’ fashion, to end with the last module as learners bring their learning to bear on a SR/MA manuscript that they write within a team.

**Table 3:**
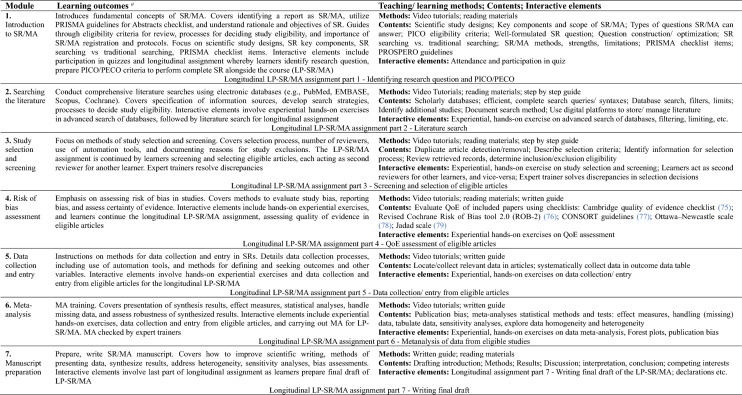
A model SR/MA online course: module, learning outcomes, and module characteristics

^a^Guided by PRISMA principles (20)

*PICO* patient, intervention, comparison, outcome, *PECO* population, exposure, comparator, outcome, *PROSPERO* international prospective register of systematic reviews, *LP-SR/MA* learner-performed SR/MA, *ROB* Risk of bias, *QoE* Quality of evidence, *CONSORT* Consolidated Standards of Reporting Trials, *SR* Systematic review, *MA* meta-analysis

Table 3 also depicts each module’s interactive elements and assessment method/s. Submitted assignments are evaluated by expert trainers, and successful completion of interactive elements in each module are prerequisites for progression to the next module.

### Alignment with PRISMA principles

Table 4 demonstrates the grounding of the course and module learning outcomes *vis-a-vis* PRISMA principles. Of note is the way preparation for module 7 (manuscript preparation) runs longitudinally across the course and builds up to bring together and culminate the knowledge elements and skills that learners acquire throughout the course.

**Table 4:**
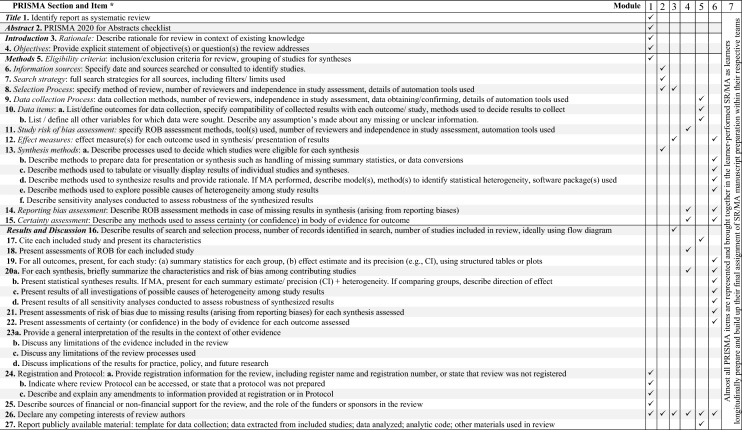
Model SR/MA online course: Alignment with PRISMA principles

^a^PRISMA items (20) described briefly due to space limitations

*ROB* Risk of bias, *CI* confidence interval, *SR* Systematic review, *LP-SR/MA* Learner-performed SR/MA, *MA* meta-analysis

### Comparison of model course with selected available online courses

The scoping review yielded three online courses: Courses 1, 2, and 3 [43–45] that were eligible for comparison to the current course. Table 5 summarizes the key features, with focus on five domains: self-pace and self-direction, interactivity, human factor, evaluation and individualized feedback on interactive tasks, and access.

**Table 5:** Comparison of model course with selected online SR/MA courses: key features and five learning and teaching domains

Characteristic	Model online SR/MA training course	Other online courses		
		1. Introduction to SR/MA: JHU [43]	2. SR/MA-Open & Free: Campbell & CMU [44]	3. Cochrane Interactive Learning: CT [45]
*Features*				
Duration	18–24 h activity + longer-term real SRMA project	Approx. 13 h	Not disclosed	> 15 h
Delivery platform	Moodle LMS	"Coursera" LMS	Open learning initiative website	Cochrane website
Learning styles	Video lectures + reading material	Video lectures + reading material	Reading material	Video lectures + reading material
Module contents	Define research question/Develop protocol; Rigorous search process; Selection criteria Critical appraisal; assess QoE; Data extraction; present findings/analysis	Selection criteria: Critical appraisal or RoB assessment; Data extraction; present findings/analysis	Introduction; Problem formulation;Search/ screen potentially eligible studies;Data extraction/coding; Introduction to effect sizes; Introduction to MA	Define research question/Develop protocol; Rigorous search process; Selection criteria; Critical appraisal/assessment RoB; Data extraction; present findings/analysis
‘Real’ LP-SR/MA project	Yes	Appears to be No	Appears to be No	Appears to be No
*Self-pace and self-direction*	Yes	Yes	Yes	Yes
*Interactivity*				
Quizzes	Introduction module	For all modules $	For all modules	For all modules $
Assignments	Full hands-on experiential for 5 modules; not for introduction module; LP-SR/MA	Limited to "framing the question" + "overview" modules, assignments based on published SRMAs ($)	No	No
Assessment methods	MCQ quizzes + Expert-reviewed and graded experiential assignments	MCQ quizzes + Peer-reviewed assignments, $	MCQ quizzes + Questions with short answers	MCQ quizzes + Questions with short answers, $
*Human factor*				
Interaction	Yes, synchronous via real-time messaging /online meetings; asynchronous via discussion forum, message boards, and email	Asynchronous interaction via discussion forum	No synchronous interaction; asynchronous interaction via email	No synchronous/asynchronous interaction
With expert trainer?	Yes	No	Asynchronous interaction via email	No
Peer interaction	Yes, via discussion forum, message boards, live messaging /meeting	Yes, via discussion forum; peer review of assignments, $	No	No
*Evaluation and individualized feedback on interactive tasks*				
Quizzes	No feedback, automated grading (all correct answers revealed after acquiring passing grade)	No feedback, automated grading, $	Automated showing of correct answer right after trainee responds, No grading	Automated feedback/show of correct answers after trainee responds, automated grading for final module quizzes
Assignments	Yes, experiential exercises; LP-SR/MA	Not in the free version, $	No	NA
With expert trainer?	1 on 1 individualized feedback from expert trainer	No expert trainer feedback. Only peer trainee review, $	No	No
*Access*				
Availability	Provide as part of higher education; Material publicly available to browse	Material publicly available to browse	Publicly available	Only 1/12 modules available as demo (introduction)
Cost	Free when provided as part of higher education including learning material and assignments grading	Subscription required for assignments grading + certificate (USD 49)	Free, but no teacher interaction, tests, college credit, or certificate of completion No subscription option	Subscription required for access to rest of course (199 British pound + VAT)

*SR/MA* systematic review/Meta-Analysis, *Univ* University; hrs: hours, *wk* weeks, *d* days, *QoE* Quality of evidence, *MCQ* Multiple-choice question, *LP-SR/MA* Learner-performed SR/MA, *SR* Systematic review, *RoB* risk of bias, *JHU* Johns Hopkins University (via Coursera), *CMU* Carnegie Mellon University, *CT* Cochrane Training, *$* requires subscription

Across these features, the proposed course stands out, with 18–24 h of activities, surpassing the durations of Courses 1 and 3 (13–15 h). A unique aspect of the model course is that it involves working on a real learner-performed SR/MA project integrated throughout the course. None of the identified courses offers this or similar feature.

As for self-paced and self-directed learning, all identified online courses incorporated it. However, our proposed course combines quizzes and assignments across all except two modules; Course 1 relies mainly on quizzes, with assignments limited to two modules only (quizzes and assignments available only with subscription); while Courses 2 and 3 have only quizzes, however, subscription is required in the former to access the quizzes, and in the latter for both the learning material and quizzes (except for the demo introduction module).

Regarding human interaction, our proposed course provides exceptionally high human interaction, integrating synchronous/asynchronous interactions with expert trainer and peer trainees via live messaging and discussion forums. Table 5 shows that the other courses provide only asynchronous interaction with peer trainees (Course 1) or expert trainers (Course 2) or no human interaction (Course 3). For evaluation, pertaining to quizzes, only the proposed course provides free automated grading; the others exhibited combinations of automated grading, or no grading (only displaying the correct answer). Regarding feedback, only our course incorporates individualized feedback from expert trainers for assignments; Course 1 offered peer-trainee feedback for assignments; Courses 2 and 3 lacked assignments overall. Regarding access, the proposed course will be free, sponsored by public funds or championing organizations.

### Learner and course evaluation methods

Table 6 depicts a variety of ways for the evaluation of the learners and the course using passive or automated data (e.g., engagement analytics generated retrieved from activity logs from training/learning platform) or actively collected data (questionnaires/interviews).

**Table 6:** Learner and course evaluation methods of a model SR/MA online course

Evaluation Method/s	Module number							Post-course
	1	2	3	4	5	6	7	
Learners								
- Attendance and participation	✔	✔	✔	✔	✔	✔		
- Interactive activities								
• Quiz (MCQ and short answer questions)	✔	✔	✔					
• Experiential exercises (pre-formulated assignments)								
Expert tutor review + feedback		✔	✔	✔	✔	✔		
• Long-term assignment (TP-SR/MA)								
Expert tutor review + feedback		✔	✔	✔	✔	✔	✔	
Course								
- Grade development in consequent attempts	✔	✔	✔	✔	✔	✔		
- Engagement analytics (activity logs from training platform)								
• Active time on training platform	✔	✔	✔	✔	✔	✔		✔
• Number of views of the learning material	✔	✔	✔	✔	✔	✔		
• Viewing of video lectures	✔	✔	✔	✔	✔	✔		
• Number of attempts required to pass modules	✔	✔	✔	✔	✔	✔		
- Critical review of long-term assignment (TP-SRMA) by tutor							✔	
- Learners’ feedback								
• Questionnaire after each module	✔	✔	✔	✔	✔	✔		✔
• Semi structured interviews								✔

*MCQ* multiple choice question, *SR/MA* systematic reviews/metanalyses, *TP-SRMA* trainee-performed SRMA

## Discussion

SR/MA offer overviews of available evidence on a topic, identify research gaps, and highlight methodological concerns [46, 47]. Critical skills for healthcare professionals include enhanced knowledge about research synthesis, including SR/MA.

The literature reveals lack of self-paced, self-directed online SR course offering experiential interactivity akin to real-life SR/MA practice, whilst offering individual feedback from expert trainer/s, where trainers and trainees possess expertise in the same field. In response, the proposed course identified such need and puts forward an interactive, and expert-guided online offering with one-on-one feedback, and subject specific assignments and exercises to enhance the depth and relevance of SR training.

As for general structure, the proposed course comprised 7 modules offering the requisite skills, concurring with similar courses [43–45]. For content, the skill sets that this course delivers included defining the research question/s and developing a protocol; rigorous search processes; selection criteria; critical appraisal; quality assessment; data extraction; and analysis and presentation of findings, all of which are essential in SR/MA [48].

Two distinctive qualities of the educational initiative proposed in the current paper, namely, self-paced and self-directed learning, are emphasized throughout the course. Self-paced learning is online learning where learners manage the pace of their learning, a flexibility that accrues the benefits of personalization, increased engagement, and improved retention [49, 50]. Self-paced learning is important in online learning, as learners may not have access to same level of support and interaction with instructors as they would in traditional classrooms [26]. Likewise, self-directed learning is active learning where trainees are accountable for their own learning by taking initiative [51]. Self-directed learning yields advantages, including student autonomy, confidence, independence, motivation, and lifelong learning competencies [52]. The proposed course is self-paced, self-directed, delivering learning materials via Moodle, allowing learners access to material at their convenience, congruent with online learning principles [53], and with three other courses we reviewed.

A third quality of the proposed course is the interactivity throughout its content. This is critical for online courses [35], and the proposed course included quizzes in the introduction and final modules, and assignments in all other modules evaluated by expert tutors providing feedback and further learning sources. Upon successful completion of module 6, learners join a virtual research team. Such undertaking directly after the course exposes learners to longer-term hands-on experiences, to write and conduct a real SR/MA. This high-level interactivity throughout the course contrasts with other online SR courses we identified, where interactivity via quizzes and peer-reviewed assignments appears limited to two modules rather than the entire course [43], or interactivity comprises only quizzes with no assignments [44, 45]. Sustained interactivity promotes student engagement, active learning, and sense of community among learners [54].

Pertaining to the ‘human’ factor, learner-instructor and learner-learner interfaces are critical for active learning [55]. However, current online courses are criticized for their lack of adequate personal communication between learners and instructors, and among learners, hindering effective learning [56, 57]. The ‘human’ factor that includes interactivity is crucial for active learning, as learner-instructor and learner-learner exchanges are imperative [58]; and such human ‘dealings’ significantly affect the satisfaction of learners [59]. Despite this, online courses might struggle to keep learners engaged and motivated and preserve sufficient social connections among learners and instructors [60].

We observed that two of the three online SR courses we reviewed did not have explicit human exchanges [43–45]. In contrast, our proposed course deliberately embraced the human factor, incorporating broad trainer-learner and learner-learner networking synchronously via real-time messaging and online meetings, and asynchronously via discussion forum, message boards, and emails. The importance of the ‘human’ factor is illustrated by research showing that while it may be possible to reduce classroom instruction time without affecting student learning, completely replacing the classroom with online instruction has negative consequences due to lack of ‘human’ factor, including lower course completion rates and worse outcomes compared to traditional classroom instruction, even when best practices for generating online discussion are followed [61]. Further support is provided by examining hybrid learning models which integrate online learning with in-person exchanges, yielding more positive results compared to fully online learning [62]. While the proposed course is not per se hybrid, however, in effect, it incorporates elements of hybrid teaching through synchronous teacher-learner and learner-learner communication/feedback.

The ‘human’ factor, particularly with a subject expert tutor in the discipline that the SR/MA is undertaken, is important in medical SR/MA, where researchers come from varied disciplines and experiences. This dictates interprofessional working in: (a) diagnosis of problem; (b) laboratory analysis; (c) clinical procedures; (d) treatment; (e) assessment of outcomes, possibly with slightly different perspectives when working together on a SR on how a given paper fits the inclusion criteria, data to be extracted, etc. If these aspects are applied to e.g., the discipline of metabolic/bariatric surgery, with its multiple interventions/procedures, laboratory, clinical and other diagnostic modalities, treatment/management approaches, and short, medium and long-term outcomes and impacts, it becomes apparent that the ‘human’ factor is critical for any proposed SR/MA courses and training. Hence, the present course leveraged the ‘human’ factor through interactive communication channels including forums, message boards, live messaging and meetings, complemented by individualized feedback from trainers after each assignment.

As regards assessments of learners’ work, evaluation and feedback are crucial. From the teachers’ side, it improves teaching planning; from the learners’ side, it enhances the learning of trainees, and promotes trainees' autonomous learning abilities [63, 64]. After examining other online courses [43–45], the proposed course is the only online SR course where expert trainers deliver individual evaluation and feedback on the assignments (e.g., related to advanced search of databases, filtering, sorting of literature; Screening for eligibility; QoE assessment; Data collection/entry). These concur with other research on enhancing e-learning experience among medical students that proposed a model that includes feedback as a critical dimension to improve the e-learning experience, emphasizing it creates positive learning experiences [65].

## Limitations

This study has limitations. A broader variety of comparable online courses would have provided broader understanding of online SR courses. More details about the content/structure of the identified courses and ability to explore subscription-based courses would have facilitated more detailed comparisons of the model course vs alternatives. Despite these limitations, the study has many strengths. It described an innovative online SR/MA course, from study design to data synthesis, through hands-on exercises and assignments rooted in a subject area. It outlined the course description, learning outcomes, module contents, interactive elements and assessments; and appraised the course alignment with PRISMA principles. We scoped the literature for online SR courses and gauged differences between them and the proposed course regarding their features and online learning characteristics (self-pace, self-direction, human factor, evaluation, feedback, access). We are not aware of other studies that undertook such tasks.

## Conclusion

This study provided insights into a pioneering model online SR/MA educational course. The course is learner-centered fostering the development of knowledge, skills, and abilities essential for success in academic and professional medical settings. Its self-paced self-directed online learning stands out for its holistic approach and innovative interactive features, including tailored experiential training to help trainees become active learners and with a human factor in terms of expert feedback provided to assist learners to solve any challenges they face. Collectively, these aspects benchmark future online courses in research synthesis methodology within medical and health-related fields. High quality SR/MA will enhance the evidence base and clinical practice.

## Supplementary Information

Supplementary Material 1.

## Data Availability

Data is provided within the manuscript.

## Abbreviations

SR/MA: Systematic reviews and meta-analyses
PRISMA: Preferred Reporting Items for Systematic reviews and Meta-Analyses
LMS: Learning management systems (e.g., Moodle)
