# Supplementary material for: Healthcare research and education: actively constructed research knowledge–a model for online systematic reviews and meta-analyses courses
Source: BMC Res Notes. 2025 Mar 10;18:103. doi: 10.1186/s13104-025-07111-8 (PMC11895192; doi:10.1186/s13104-025-07111-8)
Supplement: Supplementary file 1 — Supplementary Material 1. [file 13104_2025_7111_MOESM1_ESM.doc]

**Supplementary Table1** Objectives of the study and details of methodology for each

| **Objective** | **Methodology Details** |
| --- | --- |
| 1 | Scoping review [39, 40] of electronic databases, documents, online resources, course outlines, reports, guidelines, and other online resources was conducted about the desirable characteristics of online learning environments for effective SR/MA training |
| 2 | Course design, components and contents identified during the literature search and innovative ideas were noted, and perspectives were integrated to develop a robust online interactive, self-paced, and self-directed SR/MA model course with learning outcomes, module contents, interactive elements, and assessment methods |
| 3 | Meticulously cross-referenced the proposed course structure, modules, and contents with PRISMA guidelines [20]. Any discrepancies were addressed and refined, ensuring that the final model complied with the guidelines. |
| 4 | Current online SR/MA courses were scoped [39, 40], and their strengths and limitations appraised. Courses were selected for inclusion after reading their websites’ descriptions, and where feasible, engaging in free/demo modules to ‘feel’ the elements of the given course. Hybrid and distant learning courses were excluded. Key features of included courses were extracted (e.g., structure, content, effectiveness, user feedback), and compared with the proposed model course to incorporate best practices. |
| 5 | Potential course outcome evaluations are suggested that consider evaluating the learners and the course. |
